# Hippocampal Theta Modulation of Neocortical Spike Times and Gamma Rhythm: A Biophysical Model Study

**DOI:** 10.1371/journal.pone.0045688

**Published:** 2012-10-02

**Authors:** Eelke Spaak, Magteld Zeitler, Stan Gielen

**Affiliations:** 1 Donders Institute for Brain, Cognition, and Behaviour, Centre for Cognitive Neuroimaging, Radboud University Nijmegen, Nijmegen, The Netherlands; 2 Donders Institute for Brain, Cognition, and Behaviour, Centre for Neuroscience, Radboud University Nijmegen, Nijmegen, The Netherlands; Georgia State University, United States of America

## Abstract

The hippocampal theta and neocortical gamma rhythms are two prominent examples of oscillatory neuronal activity. The hippocampus has often been hypothesized to influence neocortical networks by its theta rhythm, and, recently, evidence for such a direct influence has been found. We examined a possible mechanism for this influence by means of a biophysical model study using conductance-based model neurons. We found, in agreement with previous studies, that networks of fast-spiking GABA -ergic interneurons, coupled with shunting inhibition, synchronize their spike activity at a gamma frequency and are able to impose this rhythm on a network of pyramidal cells to which they are coupled. When our model was supplied with hippocampal theta-modulated input fibres, the theta rhythm biased the spike timings of both the fast-spiking and pyramidal cells. Furthermore, both the amplitude and frequency of local field potential gamma oscillations were influenced by the phase of the theta rhythm. We show that the fast-spiking cells, not pyramidal cells, are essential for this latter phenomenon, thus highlighting their crucial role in the interplay between hippocampus and neocortex.

## Introduction

The hippocampal theta rhythm (3–8 Hz) and the neocortical gamma rhythm (30–100 Hz) are two prominent examples of oscillatory neuronal activity [1], [2]. The hippocampal theta rhythm is thought to reflect the “activation state” of the hippocampus [1] and is important for the temporal coordination of a variety of functions [3]–[5]. In the neocortex, cell assembly formation, a crucial prerequisite for cognitive processing, is strongly associated with gamma oscillations [6]–[8].

Both the hippocampus and the neocortex, in particular the prefrontal cortex, seem to play complementary, yet highly interdependent, roles in the formation and retrieval of memories [9]–[12]. When we take this finding into account, along with the functional importance of the theta and gamma rhythms, it is not too far-fetched to hypothesize a direct influence of the hippocampal theta rhythm on neocortical networks.

Indeed, evidence for such a direct influence has recently been found. In both awake and sleeping rats, the hippocampal theta rhythm was found to bias both the spike times of individual neurons in prefrontal cortex and the occurrence of localized neocortical gamma oscillations ([13]–[15]; see also [16]). Furthermore, in the human neocortex, the power of the “high gamma” rhythm (80–150 Hz) was found to be phase-locked to theta oscillations [17]. Importantly, this coupling between oscillations of different frequencies seems to have behavioral relevance: so far, evidence has been found to support cross-frequency coupling being involved in e.g. visual processing [18] and working memory [19].

The mechanisms by which the hippocampus is able to influence neocortical networks through its theta rhythm are not well-understood. The neuronal networks responsible for the generation of the gamma rhythm are better understood: there is quite some physiological and biophysical work available on this phenomenon [20], [21].

Interconnected networks of fast-spiking (FS) GABA -ergic interneurons with strong inhibitory chemical synapses as well as electrical synapses (gap junctions) tend to synchronize their spiking activity at a gamma frequency. Hence, they are thought to be responsible for the generation of the gamma rhythm in the neocortex [22]–[26]. Importantly, this hypothesis has been confirmed by using a direct manipulation of the activity of fast-spiking interneurons, so the involvement of these cells goes beyond mere correlation [27]. Most likely, the inhibition involved in the synchronization of such fast-spiking interneurons is of the *shunting* type [20], [28]. Shunting inhibition is a type of synaptic inhibition in which the reversal potential of the inhibitory synapse is above the postsynaptic cell's resting potential. This is different from *hyperpolarizing* inhibition, in which the reversal potential is below the resting potential. Thus, a shunting GABA -ergic synaptic event can actually be excitatory when the post-synaptic membrane potential is at or near the resting potential [20], [28].

Hippocampal efferent fibres project directly onto neurons of the prefrontal cortex [29], [30]. Both pyramidal cells and interneurons are the targets of these projections. The projections to the interneurons, however, are stronger than those to the pyramidal cells [31], [32].

Taken together, (1) the empirically observed interaction between the hippocampal theta and neocortical gamma rhythms, (2) the crucial role played by prefrontal cortex interneurons in the generation of the gamma rhythm, and (3) the preferential projection of hippocampal fibres onto these interneurons, led us to hypothesize that the fast-spiking interneurons of the neocortex are the key players in the mechanism by which the hippocampal theta rhythm influences neocortical networks. In this paper, we analyze this possibility using a biophysical model of a network of conductance-based neurons.

We briefly summarize and preview our results as follows. First, we find that networks of coupled fast-spiking interneurons are robust gamma oscillators, in agreement with previous work [20], [25], [26], [28]. Second, these interneurons impose their rhythm on pyramidal cells synaptically innervated by them. Third, hippocampal theta input to a coupled pyramidal cell/interneuron network results in theta-phase biased spike timings. Fourth, and most importantly, the frequency and amplitude of neocortical gamma oscillations are modulated by the phase of the hippocampal theta rhythm if and only if the neocortical fast-spiking interneurons receive hippocampal theta input; no such modulation is observed if only the neocortical pyramidal cells receive hippocampal theta input. Thus, we show that, indeed, neocortical fast-spiking interneurons are crucial for the coupling between hippocampal theta and neocortical gamma rhythms that is observed in experimental physiology.

## Results

**Figure 1:**
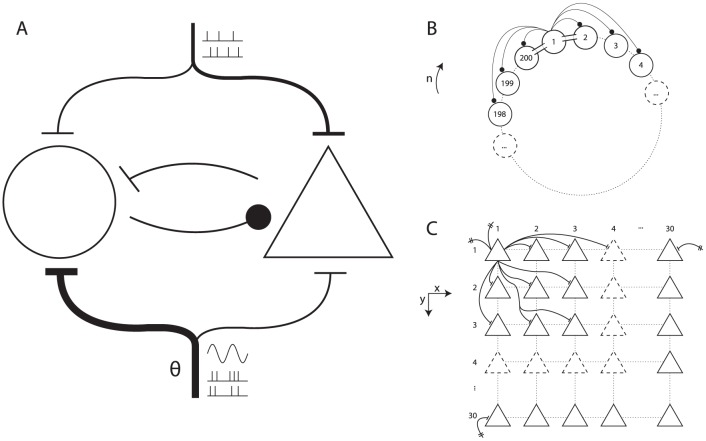
A patch of neocortex modelled by a network of interneurons coupled with a network of pyramidal cells. (A) The macroarchitecture of the model. Shown are the fast-spiking inhibitory interneuron network (circle, left), the pyramidal cell network (triangle, right), the cortical long-range afferent spike trains (top), and the theta-modulated subcortical afferent spike trains (bottom). (B) The ring-like structure of the interneuron model. Shown are some of the inhibitory synaptic connections (solid circles) and gap junctions (‘conduits’ between adjacent cells) for cell 1. (C) The two-dimensional structure of the pyramidal cell network. Shown are some of the excitatory synaptic projections from cell 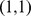 to its neighbours. Note the projections to 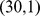 and 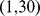 are possible because the effective distance from 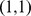 to those cells equals 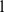 (see Methods for details).

To study the influence of hippocampal theta oscillations on neocortical spike times and gamma oscillations, we modelled a patch of neocortex by two interconnected subnetworks: one comprised of 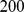 fast-spiking inhibitory interneurons (FS cells), another comprised of 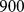 pyramidal cells (P cells). See Figure 1 for an overview of the architecture of the model.

The FS cells' membrane potential was governed according to Wang-Buzsáki equations [25]. They were arranged in a one-dimensional ring-like structure, and each cell was synaptically coupled to a subset of its neighbours, with a Gaussian probability, up to a maximum connection distance of 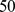 cells. Synapses within the FS cell subnetwork were GABA -ergic and shunting. Additionally, gap junctions were present between cells and their nearest neighbours. The first results reported in the present section concern only this FS cell subnetwork. Later on (as will become evident from the text), the P cell subnetwork was also involved.

The P cells were implemented as Hodgkin-Huxley model neurons (standard NEURON implementation) [33], [34]. They were arranged in a two-dimensional lattice-like structure, and each cell was synaptically coupled to a subset of its neighbours, with a Gaussian probability, up to a maximum (two-dimensional) connection distance of 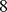 cells. Synapses within the P cell subnetwork were glutamatergic and excitatory.

The two subnetworks were coupled to each other with synapses of the same type as the ones within each subnetwork. So, each P cell received shunting GABA -ergic innervation from a subset of FS cells, and each FS cell received glutamatergic innervation from a subset of P cells.

### Gamma generation by a ring of fast-spiking cells

Networks of fast-spiking inhibitory interneurons with fast, strong, and shunting inhibitory synapses and gap junctions are hypothesized to be the main generators for the cortical gamma rhythm, and there is ample empirical evidence available that supports this hypothesis [20], [25]. In an attempt to replicate the results in the literature, we simulated the dynamics of a ring-like network of FS cells provided with a direct current input. The amplitude of the input current to the FS cells was determined by drawing a specific amplitude 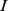 from a Gaussian distribution with mean 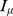 and a standard deviation determined by a coefficient of variation over cells 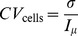. A high 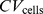 results in different cells in the network having a highly heterogeneous input. This way of determining input current amplitude allowed us to study the effects of both net input drive and drive hetereogeneity on the synchronization properties of our network.

**Figure 2:**
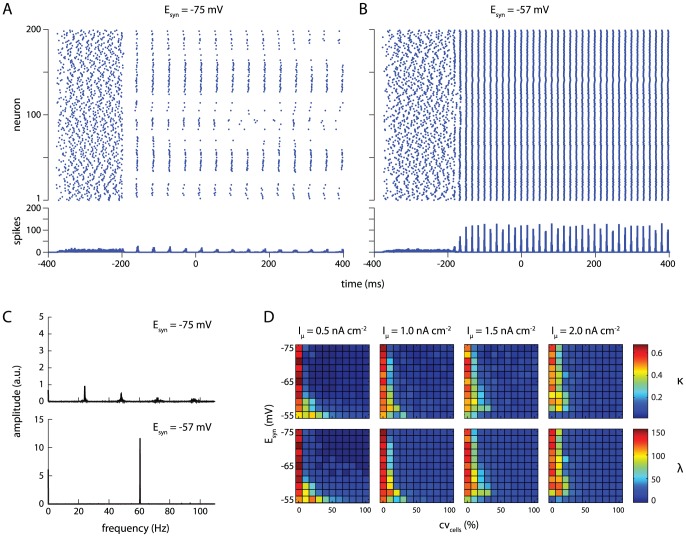
Shunting inhibition increases robustness in a network of fast-spiking inhibitory interneurons. (A–B) Rasterplots (top) and spike histograms (bottom) for simulations GABA -synaptic reversal potentials of 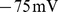 (A) and 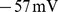 (B). For these plots, mean drive 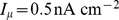, drive variation 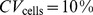. Synapses were activated at 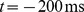; plots are truncated at 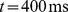. (C) Amplitude spectra for spike histograms. Spectral analyses were performed on complete histograms, ending at 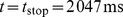. (D) Two measures of network synchronization, network coherence 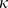 (top row) and average spike volley peak height 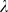 (bottom row), as a function of drive variation over cells 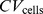 (x-axis), synaptic reversal potential 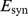 (y-axis), and mean drive 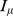 (separate columns). Shunting values of 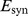 result in stronger synchronization with increasing drive heterogeneity.

When the amplitude of the direct current input is small (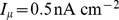) and for a moderate amount of variation over cells (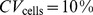), networks in which the GABA -synaptic reversal potential was hyperpolarizing (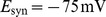) show only weak synchronization (Figure 2A). Shunting GABA -synapses (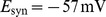), on the other hand, result in a strongly synchronized network (Figure 2B). Additionally, for shunting inhibition, nearly all cells in the network fire exactly once per gamma cycle, while the hyperpolarizing reversal potential of 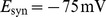 causes the strongly excited cells to silence out the more weakly excited ones [35]. Fast-spiking interneurons firing once per gamma cycle, as we observe in our shunting inhibition condition, is in agreement with observations *in vitro* [36], *in vivo* [37]–[39] and previous modelling work [25], [40].

Spectral analyses of spike histograms revealed only a moderate peak near 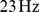 (and higher harmonics) for a synaptic reversal potential of 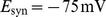 (Figure 2C, top panel), while a strong peak at the gamma frequency of 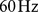 appears for a synaptic reversal potential of 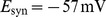 (Figure 2C, bottom panel).

To assess the effect of the synaptic reversal potential on network synchronization in a more general sense, we employed two different measures of network synchrony: network coherence 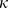 ([25]; see equation 9) and average volley peak height 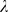 (see equation 11). As expected, these yield highly similar results, as revealed by correlation analysis (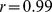; 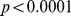). 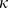 and 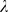 were consistently high with homogeneous drive (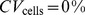; Figure 2D, first column within each plot), and decreased with increasing heterogeneity (Figure 2D, x-axis of plots).

As reported previously [28], the rate of decrease with heterogeneity was dependent upon the reversal potential of the GABA -ergic synapses. Specifically, with a small drive of 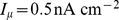, coherent oscillations (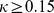) only occurred at heterogeneity levels of 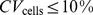 when synapses were hyperpolarizing (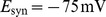), while shunting inhibition (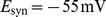) resulted in coherent oscillations up to 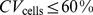 of heterogeneity (Figure 2D, left).

Interestingly, and to our knowledge not previously reported, the most robust synaptic reversal potential (i.e., the value of 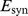 that results in coherent network synchronization up to the highest level of drive heterogeneity) changed when the amplitude of the input current was varied: for a drive of 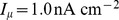, robust oscillations (up to 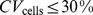) still occurred at 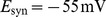. When the drive was increased further to 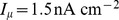, network synchronization was most robust at 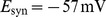 (up to 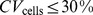; Figure 2D, third column of plots). At 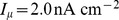, the optimal reversal potential for synchronization was 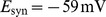 (synchronization up to 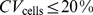; Figure 2D, right-most column of plots).

The optimal reversal potential seemed to continue to shift for even higher values of 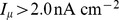, but, starting at 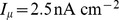, the fraction of active cells in the network and dependence of network coherence on drive variation started to decrease markedly (data not shown).

### Pyramidal cell network gamma oscillations by interneuron shunt

The previous section showed that gamma synchronization does indeed occur in the ring of coupled fast-spiking inhibitory interneurons in our model. The next step is to show that this mechanism is sufficient to drive gamma oscillations in a network of pyramidal cells.

To address this question, we investigated the behavior of a network of 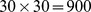 pyramidal cells. Each P cell was synaptically coupled to, on average, 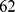 pyramidal cells. Additionally, the P cells received incoming synapses from simulated fibres carrying Poisson spike trains, modelling cortical background input. P cells projected to the fast-spiking cells with glutamatergic synapses. Each pyramidal cell received a variable number of incoming GABA -ergic synapses from the fast-spiking cells. These GABA -ergic synapses were of the shunting type.

**Figure 3:**
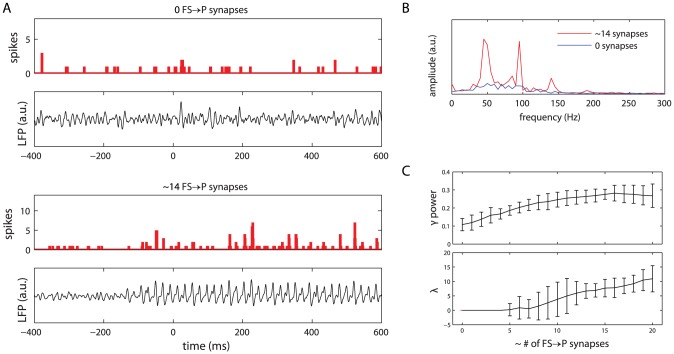
Pyramidal cells show gamma-synchronized activity when cells receive shunting inhibition. (A) Spike histograms and simulated LFP traces for the unconnected (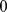 FS to P synapses per P -cell) and connected (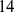 FS to P synapses per P -cell) conditions. Other input to the P -cells consisted of constant-rate Poisson spike trains. (B) Amplitude spectra for the simulated LFP in the connected (red) and unconnected (blue) conditions. The LFP spectrum for the connected condition shows a clear increase in power in the gamma band (30–80 Hz). (C) Relative gamma band power (top) and pyramidal cell network synchronization (bottom) as a function of the average number of GABA -ergic projections from the FS cells to a single P -cell. Relative gamma power increases steadily with the number of synapses, reaching a maximum at 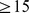 synapses per P -cell. Network synchronization starts to occur at 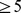 synapses per P -cell. Shown are the mean values for 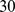 simulation runs; error bars represent 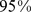 confidence interval.

First, we analyzed the spike times of the pyramidal cells. When the pyramidal cell subnetwork only received constant-rate Poisson spike train input and did not receive shunting inhibition from the fast-spiking cells, a non-synchronized activity pattern was observed (Figure 3A, top spike histogram). When the fast-spiking cells projected to the pyramidal cells, however, the latter tended to synchronize in a gamma rhythm (Figure 3A, bottom spike histogram). The gamma synchronization of the pyramidal cells involved a much smaller portion of cells than that of the fast-spiking cells: for the simulation shown, the maximum proportion of cells active in a single 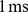 time window was 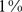 for the pyramidal cells and 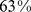 for the fast-spiking cells (compare Figures 2B, bottom spike histogram, and 3A, bottom spike histogram).

Apart from the spike times, shown in the histograms, we also analyzed the electrical activity of the pyramidal cells, as measured by the simulated local field potential (LFP ; see equation 12). The simulated LFP traces are shown in Figure 3A (panels below the spike histograms). The simulated LFP shows irregular oscillations for the condition in which the pyramidal cells do not receive shunting inhibition from the fast-spiking cells (Figure 3A, top panel). However, when the GABA -ergic projections from the FS -cells to the P -cells are active, the simulated LFP shows a regular oscillatory pattern (Figure 3A, bottom panel).

This effect is evident even more clearly from the LFP amplitude spectra (Figure 3B): the spectrum for the connected condition (red) shows a marked increase in gamma-band power over the unconnected condition (blue). In particular, a peak near 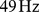 can be observed. Note that this frequency is somewhat different from that reported for the interneuron-only simulations (previous section). This is because the net total input to the interneuron subnetwork was somewhat different for the present analyses (i.e., Poisson spike train input) than it was for the interneuron-only simulations (i.e., direct current input). The results presented in the previous section were based on a direct current input because that allowed a better comparison with the work of Vida et al. [28].

To determine the amount of GABA -ergic shunting needed for the pyramidal cells to synchronize in a gamma rhythm, we systematically varied the average number of projections from the fast-spiking interneuron subnetwork to each pyramidal cell. The relative power in the gamma band (30–80 Hz) of the simulated LFP steadily increased with the number of synapses, and reached a plateau at about 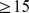 synapses per cell (Figure 3C, top panel). The network remained in an unsynchronized state for up to 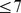 synapses per cell, and network synchronization increased steadily above this threshold (Figure 3C, bottom panel).

The continuity in rise of the relative gamma power with respect to the number of synapses, when contrasted with the initial zero-valued plateau that was observed for the synchronization, is explained by the fact that the LFP is primarily a measure of post-synaptic potentials (PSP s). For a small, but non-zero connection strength between a network with synchronized firing at a gamma frequency (i.e., the FS cells) that projects to another network of cells (i.e., the P -cells), the PSP s will also oscillate, albeit with a small amplitude, in the gamma frequency range. If the oscillatory PSP activity is small and subthreshold, the oscillatory input may not be reflected in the spiking activity and, consequently, not be observed as network synchronization, unless the synchronized PSP s are sufficiently strong relative to the cells' firing threshold.

### Theta modulation of spike times

To investigate the influence of ascending hippocampal fibres, carrying a theta rhythm, on the spike times of neocortical fast-spiking and pyramidal cells, we supplied our model network with variable-rate Poisson spike trains, in addition to the constant-rate background input already present. These ‘theta fibres’ varied their firing frequency according to a sinusoid oscillating at a theta frequency of 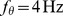 (see equation 8; this particular frequency was chosen to correspond to observed theta frequencies in anaesthetized rats [13]. Note that we will use the term ‘theta fibres’ to refer to the projections from the theta-modulated Poisson spike trains to the cells of our model network. We do not wish to imply that there are anatomically identifiable fibres running from the hippocampus to the neocortex that are dedicated exclusively to the propagation of the theta rhythm.). Each fast-spiking cell received, on average, 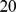 incoming synapses from these variable-rate fibres, while each pyramidal cell received, on average, 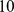.

**Figure 4:**
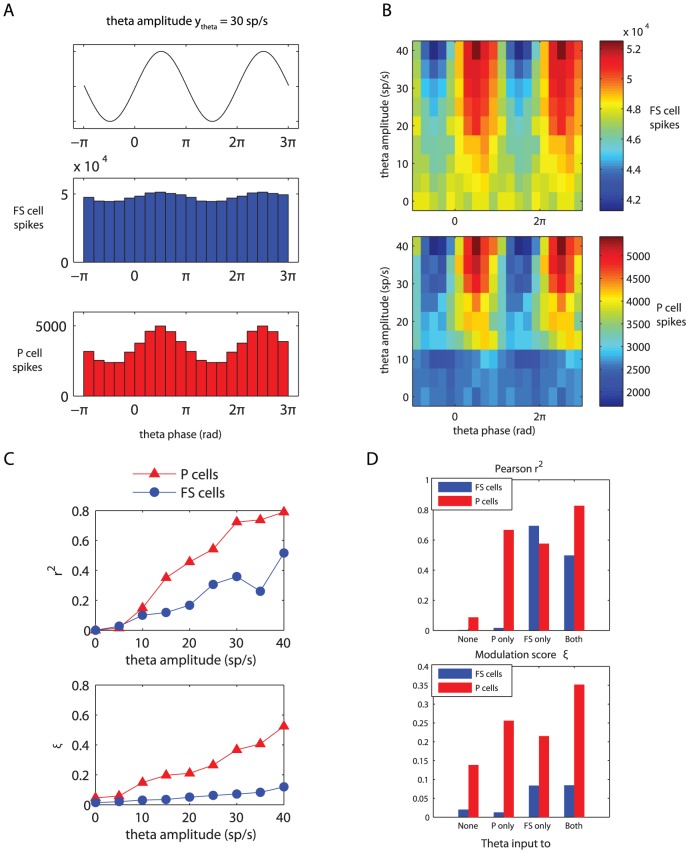
Theta modulation of fast-spiking and pyramidal cell spike times. Fast-spiking cells received, on average, twice as many theta-modulated input synapses as the pyramidal cells, which, relative to the total input, is four times as many (see main text for details). (A) Spike histograms for the fast-spiking (blue, middle) and pyramidal (red, bottom) cells relative to theta phase. The amplitude of the incoming theta rhythm was set to 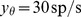 (see equation 8). Histogram bin size is 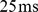. Above the spike histograms, the ‘raw theta signal’ (see equation 14) is plotted, to show which phases in the theta cycle correspond to peaks and troughs. (B) Spike counts (color code) relative to theta phase (x-axis) for the fast-spiking (top) and pyramidal (bottom) cells as a function of theta amplitude 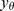 (y-axis). Spike count bin size is 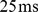 and bins were non-overlapping. (C) Two different measures of theta modulation of spike times: Pearson's 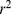 (top) and relative modulation amplitude 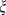 (bottom; see equation 15) as a function of incoming theta amplitude 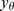. (D) The same two measures as a function of theta input distribution.

An analysis of the spike times of the fast-spiking and pyramidal cells revealed that the number of spikes (in 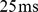 bins) occurring at the peak of the theta rhythm is larger than the number of spikes occurring at the theta trough (Figure 4A). This finding holds for both cell populations, and is easily explained by noting that the total net excitation experienced by a cell is higher during a theta peak than during a theta trough. (The role of ‘coincidence detection’-like phenomena in the increased number of spikes during theta peaks is probably negligible; the P cell membrane time constant is around 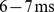.) Furthermore, theta modulation of spike times increases with increasing theta input amplitudes (Figure 4B).

### Differential modulation of pyramidal and fast-spiking cells

The theta modulation of pyramidal cells was much stronger than that of the fast-spiking cells (Figure 4A and B). This finding is remarkable, since the fast-spiking cells receive, on average, twice as many incoming theta-modulated synapses as the pyramidal cells (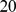 vs. 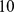), and even four times as many when the ratio of theta-modulated to constant-rate fibres is taken into account (FS: 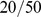; P: 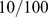). The resulting differential theta-modulation is in accordance with physiological findings: Sirota et al. [13] found that, in rats, the spike times of neocortical/hlpyramidal cells are more strongly biased by the hippocampal theta rhythm than the spike times of neocortical fast-spiking interneurons.

In order to quantify this difference, we computed two measures of theta modulation: Pearson's correlation coefficient 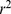, computed between the raw theta signal and the aggregated spike histograms; and our modulation score 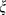, as described by equation 15. Indeed, plots of these measures confirm that the theta modulation of the pyramidal cells is consistently larger than that of the fast-spiking cells (Figure 4C). For a theta modulation amplitude of 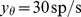, the parameter setting used for Figure 4A, the relevant values were 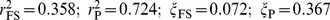.

**Figure 5:**
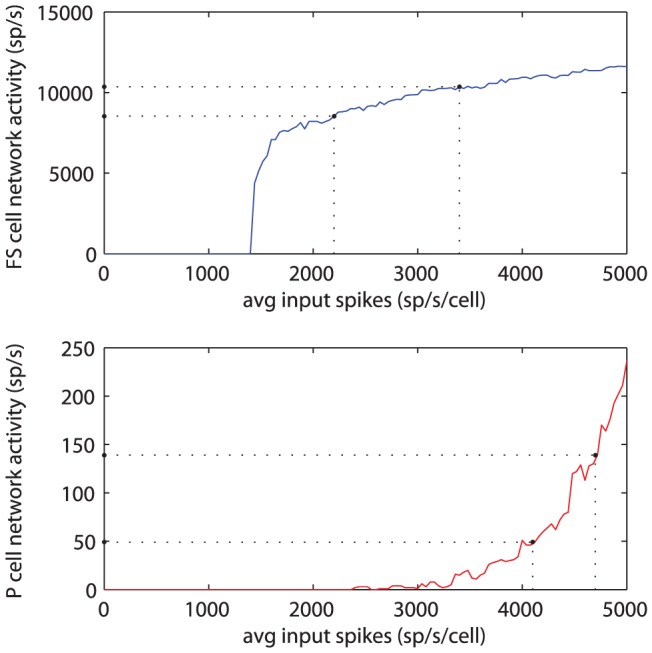
Frequency/input curves for the two model subnetworks: the fast-spiking cells (top) and the pyramidal cells (bottom). The dotted lines perpendicular to the x-axis represent the total range of spike train input experienced by the subnetworks due to the theta-modulated input fibres. Dotted lines perpendicular to the y-axis represent the resulting subnetwork spike activity. While the theta-modulation of input spikes is stronger for the FS cells than for the P cells, the resulting difference in subnetwork spike activity is greater for the P cells, as can be observed from the intersection of the horizontal lines with the y-axis.

To find an explanation for the much stronger theta-modulation of spike activity of the P cells, when compared to the FS cells, we investigated the firing characteristics of both of our subnetworks in response to different levels of Poisson spike train input arriving at a single cell. Results for this simulation are shown in Figure 5. The FS cells show a reasonably flat response to increasing input after an initial strong rise, whereas the P cells show a very steep, exponential response. Given the mean theta input fibre spike rate of 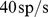, for a theta input amplitude of 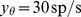, the spike rate of the theta-modulated input fibres varies between 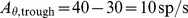 and 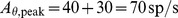. Consequently, the total input (the sum of the constant-rate input and the theta-modulated input) experienced, on average, by a single FS cell varies between 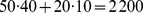 and 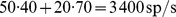. The average total input to a single P cell varies between 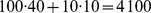 and 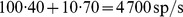. These values correspond to the vertical lines in Figure 5. Notice the relatively much larger y-axis range corresponding to these input values for the P cells, when compared to the FS cells. This explains why the theta-modulation of P cell activity is so strong, even though the FS cells receive a much more strongly theta-modulated input.

### Effect of theta input to different subnetworks

In the above-mentioned simulations and analyses, theta-modulated input, when present, was always presented to both subnetworks of P and FS cells of our model network. We also investigated the effect of theta-modulated input to each of the subnetworks separately.

To assess the amount of theta-modulation present in the spike times of the FS and P cell subnetworks, we computed two measures sensitive to this modulation (see Methods). Figure 4D shows the results for these simulations. The spiking activity of the pyramidal cells is theta-modulated when either (or both) of the two subnetworks receives theta-modulated input. The spiking activity of the fast-spiking cells, however, is only theta-modulated when they directly receive theta-modulated input, irrespective of whether the pyramidal cells receive theta-modulated input or not.

This finding can be explained by looking at the input response curves for the two subnetworks (Figure 5): (1) the FS cells show a flat input response curve, thereby requiring a large change in input to obtain a small change in activity, and (2) the overall spiking activity of the P cells is very small, when compared to the FS cells or the input spike trains. When only the P cell subnetwork receives theta-modulated input, this will be reflected in the spiking activity of this subnetwork (see Figure 4D). The P cell subnetwork activity is still very low, however, when compared to the total input to a single FS cell, coming from other FS cells and input spike trains. Therefore, the theta-modulated P cell activity will not have a large enough impact on the FS cells to be noticeable in their spiking activity.

### Theta modulation of LFP gamma activity

In the previous section we demonstrated that the spiking activity of the pyramidal and fast-spiking cells is theta-modulated when these subnetworks are presented with theta-modulated input spike trains. Additionally, we showed that pyramidal cell activity is theta-modulated if either the pyramidal cells or the fast-spiking cells receive theta-modulated input, while fast-spiking cell activity is only theta-modulated when they directly receive theta-modulated input.

This raises the following two interrelated questions. First, is the LFP gamma activity, caused by the FS cell shunting inhibitory synapses on the P cells, also theta-modulated? And second, if a theta/gamma coupling exists, which theta-modulated input fibre projections are necessary and, therefore, presumably responsible, for this coupling?

#### Gamma frequency modulation by theta phase

**Figure 6:**
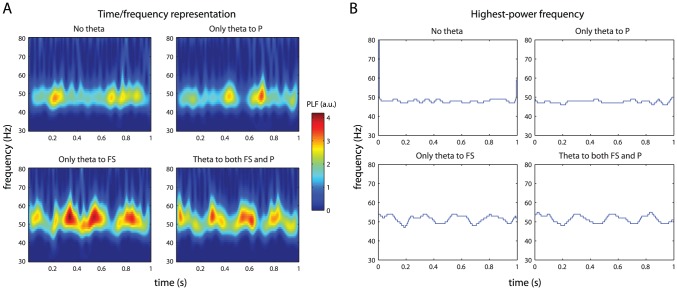
Effect of theta-modulated input on LFP gamma frequency. (A) Time-frequency representations of one second of simulated LFP activity. The four panels correspond to theta-modulated input fibres projecting to different parts of the model network. (B) The frequency within the gamma band with the highest power, for the same data segments shown in A. Theta phase/gamma frequency coupling is observed if and only if the FS cells receive theta-modulated input.

To answer these questions, we computed simulated LFP from our model network in four different conditions: no theta input, theta input only to the P cells, theta input only to the FS cells, or theta input to both subnetworks. Time-frequency representations for 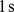 of these recordings are shown in Figure 6A. In all four conditions the spectral power peaks in the gamma range near 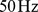.

To determine whether a phase/frequency coupling between the incoming theta signal and the gamma LFP occurred in our network, we plotted the highest-power frequency from the above-mentioned time-frequency representations as a function of time (Figure 6B). These plots show that the highest-power gamma frequency varied with the theta rhythm only when the FS cells received theta-modulated input. Variation in the peak gamma frequency as a function of theta phase thus requires projections from the simulated hippocampal afferent fibres to the FS cells.

#### Gamma amplitude modulation by theta phase

**Figure 7:**
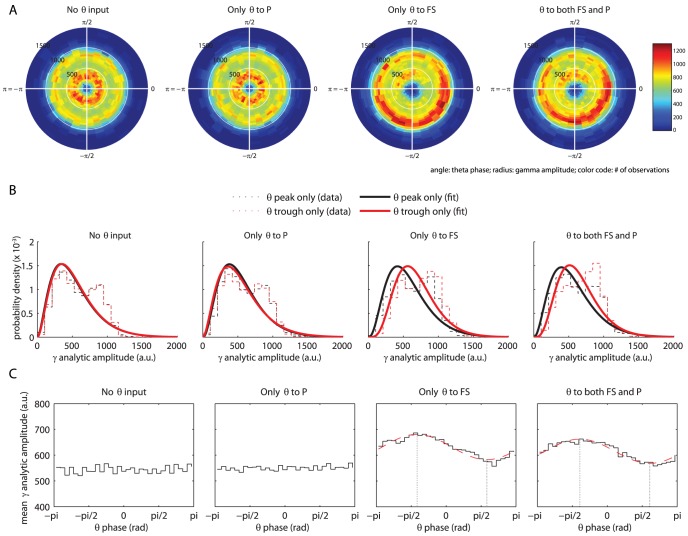
Effect of theta-modulated input on LFP gamma amplitude. (A) The distribution of a composite theta phase/gamma amplitude signal 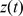 in the complex plane. Color code represents the number of observations; angle corresponds to theta phase (divided into 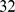 equally-sized bins); radius corresponds to gamma amplitude (divided into 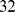 equally-sized bins). (B) Histograms of gamma amplitudes occurring in 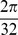 wide phase bins centered at the peak of the theta rhythm (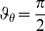; black) and at the trough (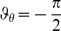; red). Dashed lines correspond to the observed data histograms; solid lines represent the best-fit gamma distribution for this data. When FS cells receive theta-modulated input, the best-fit distribution for the theta trough is shifted to the right, compared to the distribution for the theta peak (parameter differences are significant, 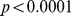). (C) Mean gamma amplitude as a function of theta phase. For the two rightmost plots, the best-fit sine functions are shown by a dashed red line. Gamma amplitudes are higher at the trough than at the peak of the theta rhythm, and the lowest and highest gamma amplitudes (indicated by dotted vertical lines) occur somewhat after the theta peak and trough, respectively.

To determine whether the gamma amplitude is modulated by theta phase, we computed a composite theta phase/gamma amplitude signal 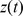. Theta phase is distributed uniformly, since the theta rhythm is generated by a simple sine function. Therefore, clustering of 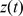 in the complex plane (resulting in a radially asymmetric distribution around the origin) is an indication of phase/amplitude coupling [17] (see Methods for details). Distributions of 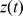, for each of the four theta input conditions, are shown in Figure 7A. 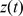 is distributed uniformly with respect to theta phase when only the P cells receive theta-modulated input. However, when the FS cells receive theta-modulated input, the distribution is clearly non-uniform.

To get a more detailed view of the nature of theta phase/gamma amplitude coupling, we investigated the distribution of gamma amplitudes 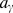 at just the peaks and troughs of the theta rhythm (the phase relates to the sine phase, so a peak is at 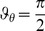 and a trough is at 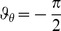). Figure 7B shows these distributions for each of the four theta input conditions. Note that the data histograms in Figure 7B do not directly correspond to a single ‘arm’ of the polar phase/amplitude histograms shown in Figure 7A. To obtain the distributions most accurately corresponding to the two theta extremes, bins for 7B were *centered* at either the theta peak or trough, while the theta peak and trough correspond to a bin *edge* for 7A. The solid lines in this figure correspond to the best-fit gamma distributions of gamma amplitudes for the theta trough (red) and the theta peak (black). We used gamma distribution fits for our amplitude data because we wanted to be able to compare our results with Canolty et al. [17]. The distribution we observed, however, seems to correspond more to a bimodal distribution (also visible as two concentric rings of higher distribution density in all four plots of Figure 7A). The best-fit distribution is shifted to the right, compared to the distribution for the theta peak, when the FS cells receive theta-modulated input. The differences between the distribution parameters are significant (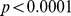). This indicates that gamma amplitude is higher at the theta trough than at the theta peak; a finding that is in agreement with physiological findings in humans [17].

Finally, to get a quick ‘bird’s eye' view of the coupling between theta phase and gamma amplitude, we divided theta phase into 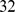 bins and computed the mean gamma amplitude for each of these bins. Plots of these data (Figure 7C) clearly show that, when the FS cells receive theta-modulated input, gamma amplitudes are higher at the trough than at the peak of the theta rhythm. The lowest and highest gamma amplitudes occur somewhat after the theta peak and trough, respectively. More specifically, the extremes in gamma amplitudes lag behind the (opposite) theta extremes by 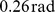 (for the FS only condition) or 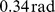 (for the FS+P condition). These values correspond to delays of 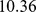 and 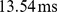.

### Robustness of core results to various model parameters

So far, we have used a fixed theta frequency of 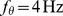 throughout our paper, which is on the low end of the frequency range usually associated with theta oscillations [see, e.g., 41]. To determine whether the results we obtain are specific to 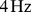, or whether they generalize to other theta-range frequencies, we systematically varied the frequency 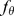 between 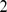 and 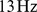 and repeated our phase/amplitude analysis based on the composite signal 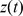. Distributions of 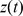 for the varied theta frequencies are shown in Figure S1. No difference in 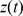 distribution is apparent for the different theta frequencies. We quantitatively verified this by computing 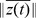, i.e., the norm of the mean of all composite phase/amplitude values. We examined whether this value depended on the frequency of the theta input (Figure S2). Although we observed a correlation trend between these two variables (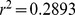), this trend did not reach significance (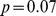). We conclude that our main finding does not crucially depend on theta frequency. Electrophysiological data suggests that the projections from the hippocampus to neocortical networks are stronger to interneurons than to pyramidal cells [31], [32]. We incorporated this difference into our model by supplying the FS cells with a more strongly theta-modulated input than the P cells, yielding the results described above. We highlighted the crucial role the FS cells play in the coupling between the theta and gamma rhythms. An additional simulation, in which we switched the two theta-modulated input strengths (i.e., in which the input to the P cells was more strongly theta-modulated than the input to the FS cells), resulted in the same outcome: a coupling between the theta and gamma rhythms only occurred in the two conditions in which the FS cells received theta-modulated input (results not shown). Therefore, the crucial role played by the FS cells is not dependent on them receiving more strongly theta-modulated input than the P cells. This, of course, prompted the question: what amplitude of theta modulated input *would* drive the P cell gamma activity to become theta modulated, in the total absence of theta input to the FS cells? To gain insight into this question, we disconnected all theta fibres from the FS cells, while making no other changes to the model (so FS to P synapses were intact, etc.). We varied the mean spike rate of the theta input fibres between 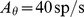 and 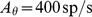, while the amplitude of the modulation was set to 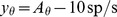, so we simultaneously varied both the average spike rate of the theta fibres and its modulation strength. We analyzed theta/gamma phase-amplitude coupling again by computing the mean of 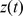, as described above. Results for this analysis are shown in Figure S3; the dashed horizontal line indicates the value of the plotted coupling metric for the physiologically constrained parameters used in the original simulations described in the bulk of this paper (this parameter value is indicated by the dotted vertical line). It can clearly be seen that the amount of theta modulation required for theta/gamma coupling, when only the P cells receive theta input, is much higher than when the FS cells receive theta input as well. Specifically, coupling comparable to the results described above (obtained with 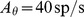, 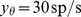, modulation of 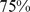 of mean) is only observed with the very high values 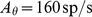 and 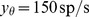 (modulation of 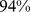 of mean) or above, when theta input is restricted to the P cells only. A further interesting factor to investigate is to what extent does the observed theta/gamma coupling depend on the gamma synchronization of the FS cells? Since we found that gamma synchronization cruciallly depends on the value of the GABA -ergic synaptic reversal potential 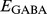, we varied this parameter to investigate this question. We computed, for various values of this parameter, the correlation between the sinusoidal theta rhythm used as input to the model network, and the amplitude envelope of the gamma-filtered LFP. (Note that this correlation measure is somewhat different from the mean composite phase/amplitude vector length we report above. We use a correlation here instead because binning the gamma amplitude values is not well-defined when gamma synchrony is very low.) Results for this analysis are shown in Figure S4. Evident from this plot is a clear regime between about 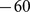 and 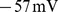 where theta/gamma coupling occurs, whereas for both lower and higher GABA reversal potentials, the coupling is absent. These values of 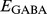 correspond well to the values which were found to lead to the greatest robustness in gamma synchronization of the FS cells (see Figure 2 and earlier in Results). Finally, we note that our choice of simulated LFP potential is not trivial. Often, the sum of synaptic transmembrane currents is used to model the LFP . The LFP indeed mainly depends on postsynaptic currents, but it is mainly the low-frequency components of these currents that determine the extracellulary measured LFP [42]. In effect, the extracellular medium acts as a rather complex low-pass filter. While attempts have been made at accurately computing the LFP , taking this into account [42], [43], we decided to use the summed membrane *potential* as a simple proxy instead. The temporal profile of the membrane potential is also low-pass filtered when compared to the transmembrane currents, due to the conductance properties of the membrane. Of course, it is important to ensure that our core results do not qualitatively depend on the choice of our simulated LFP signal. Therefore, we conducted a control analysis in which we investigated the effect of the choice of simulated LFP on our results (the composite phase/amplitude distribution). Results for this analysis are shown in Figure S5. The distribution is quite a bit sharper when considering currents (right plot) instead of potential (left plot, for reference). This is expected, because the ‘smearing’ properties of the membrane potential are not incorporated for the right plot. Crucially, however, the main features of the distribution remain the same: a clustering is observed for gamma amplitude as a function of theta phase.

## Discussion

We have put forward a biophysical conductance-based neural network model, composed of fast-spiking (FS) and pyramidal (P) cells, that displays robust gamma oscillations. When supplied with hippocampal theta-modulated input fibres, the network shows spike activity that is biased by the theta rhythm. Furthermore, both the frequency and amplitude of the local field potential (LFP) gamma oscillations are modulated by the phase of the theta rhythm. However, this latter phenomenon *only* occurs when the FS cells directly receive hippocampal theta-modulated input, highlighting the crucial role these cells play in the interplay between neocortex and hippocampus.

The conductance-based neuron models we used in our study provide the most realistic approximation of actual single-neuron activity [44] to date. This is in sharp contrast to integrate-and-fire neurons, which can be simulated far more quickly, but provide a much rougher approximation of neural activity [44]. Cross-frequency coupling of neuronal oscillations has been analysed with biophysical model neurons before, mainly in relation to network firing rate. Mazzoni et al. [45], for instance, find a significant coupling between delta phase and gamma amplitude, for network parameters quite similar to the ones we used in the present study. Crucially, previous work on this topic has generally used integrate-and-fire neurons, whereas we show cross-frequency phase/amplitude coupling for the–to our knowledge–first time using a conductance-based model for all simulated neurons.

### Gamma synchronization and oscillations

When studied in isolation, our subnetwork of FS cells, coupled with fast inhibitory synapses and gap junctions, shows synchronized activity at a gamma frequency (Figure 2). For GABA -ergic synapses with a hyperpolarizing reversal potential, this synchronization quickly dissipates with increasing heterogeneity of the input drive. For shunting reversal potentials, however, the network remains robustly synchronized for relatively high levels of drive heterogeneity. This finding is a replication of earlier work [20], [28].

The optimally robust synaptic reversal potential for the gamma synchronization of the FS cells was dependent upon mean input drive (Figure 2D). For increasing input currents, the optimal reversal potential shifts towards more negative values, but remains well above the membrane potential at rest. This phenomenon likely reflects the important role of strong mutual inhibition between the FS cells in the generation of the gamma rhythm [25]. When the input current is strong, the FS cells become strongly excited, and the inhibition balancing this excitation may not be strong enough when the synaptic reversal potential is relatively high. This might allow some cells to fire out of phase with the gamma rhythm. Lower synaptic reversal potentials, on the other hand, result in sufficiently strong inhibition to silence out any ‘rogue’ cells during the interval between population spike peaks.

The FS cells proved effective in imposing their synchronized rhythm on a population of P cells (Figure 3). When the FS cell subnetwork was coupled to the P cell subnetwork, the LFP quickly became dominated by the GABA -ergic post-synaptic potentials caused by the FS cells' spiking activity. This resulted in an increase in LFP power in the gamma band and in an increased synchronization of the P cells' spike times. Thus, gamma oscillations in our inhibitory interneuron subnetwork carry over to the pyramidal cells to which it was coupled. This fits nicely with prominent theories on the role of the gamma rhythm as a fundamental computational mechanism [37], in which the amplitude of input to a pyramidal cell network can be transformed into a phase code. This transformation is achieved through a coupling of the network to fast-spiking interneurons network which are firing in a gamma rhythm [37].

In *in vivo* recordings of cortical LFP , the power spectrum is usually characterized by a very dominant 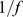 or ‘power law’ component [46]. We do not show such a power law scaling in the spectra computed for our model (Figures 2C, 3B, 6A), which is not in line with these *in vivo* findings. However, the power law scaling of LFP power is usually absent from *in vitro* recordings [e.g. 36], [47], [48]. Therefore, the involvement of large-scale dynamic interactions with the rest of the brain seems essential for the power law scaling to be apparent. Since model studies typically simulate a small patch of cortex, their object of study is much more similar to *in vitro* than to *in vivo* recordings. This is also the case for our study, so the lack of a dominant 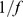 component is expected for our power spectra.

### Effect of theta-modulated input

When the network received not only constant-rate input, but also input from theta-modulated hippocampal afferent fibres, its spiking activity was modulated by the theta rhythm (Figure 4). This is in accordance with empirical findings [13]–[15].

Theta-modulated input to our model network also had an effect on the gamma oscillations visible in the LFP . Both the frequency (Figure 6) and the amplitude (Figure 7) of the gamma oscillations were modulated by the phase of the theta rhythm, but only when the FS cells directly received a theta-modulated input. Theta/gamma coupling was completely absent, both regarding frequency and amplitude, when only the P cells received theta-modulated input, even though the P cells' spiking activity *was* theta-modulated in this condition. This can be explained by the fact that the gamma rhythm is generated by the FS cells and imposed upon the P cell subnetwork by these cells. It follows easily that, when the activity of the FS cells is not theta-modulated, the gamma rhythm, as observed in the LFP , is not theta-modulated either. An exception to this rule was only observed when theta-modulated input to the P cells was much higher than would be physiologically plausible (Figure S3).

The frequency of the gamma rhythm was higher during the peak of the theta rhythm than during the trough (Figure 6). The gamma rhythm is generated by the FS cells. During the theta peaks these experience a stronger net total input and, therefore, increase their firing rate while remaining synchronized, hence resulting in a higher frequency of the corresponding gamma oscillations. We do not know of any empirical reports on a coupling between hippocampal theta phase and neocortical peak gamma frequency, but this is an interesting prediction that can be tested.

The amplitude of the LFP gamma oscillations was also influenced by the phase of the incoming theta rhythm (Figure 7). Gamma amplitude was higher during the trough than during the peak of the theta rhythm, a phenomenon that was also reported for human subjects [17]. The highest gamma amplitude occurred somewhat after the theta trough and the lowest gamma amplitude occurred somewhat after the theta peak.

The mechanisms by which the amplitude of gamma oscillations is actually *highest* during the periods of *least* net total input to the network are not entirely clear to us. Canolty et al. [17] found a similar phase/amplitude coupling between the theta and gamma rhythms, in the human neocortex. As to the mechanism of this coupling, they hypothesize that “basal forebrain cortical-projecting GABA ergic neurons (…) preferentially synapse onto intracortical GABA ergic neurons (…), with disinhibitory spike bursts causing a brief increase in gamma power at the theta trough” [17, p. 1628].

While the basal forebrain (BF) GABA -ergic neurons probably play a role in the modulation of neocortical networks [49], our results suggest that they are not responsible for the coupling between theta phase and gamma amplitude. We were able to reproduce such a coupling using only *excitatory* (glutamatergic) fibres to carry the theta rhythm. Of course, this does not directly preclude the alternative hypothesis (i.e., the BF GABA -ergic neurons playing the crucial role), but we believe this alternative to be unlikely for the following reason. The gamma oscillations visible in the EEG and LFP are mainly due to post-synaptic potentials in the pyramidal cells resulting from spike events in intracortical GABA -ergic fast-spiking cells. If the latter cells receive inhibition (e.g., from BF GABA -ergic neurons), the result would be a *weakening* of the gamma oscillations, and not an increase in amplitude. Therefore, we believe that excitatory, rather than inhibitory, projections to the FS cells are responsible for the modulation of gamma amplitude by theta phase.

### Functional role of cross-frequency coupling

Between- and within-region coupling between neuronal oscillations of different frequencies, in particular between the phase of a lower-frequency oscillation and the amplitude of a higher-frequency one, has been observed in rodents [13]–[15], macaque monkeys [50], and humans [17]–[19]. This type of cross-frequency coupling is thought to play an important role in the integration of the various timescales at which the brain processes information: large-scale dynamical brain networks, reflected in lower-frequency oscillations, entrain high-frequency local neuronal computations, thereby enabling various brain regions to process the incoming information in the most efficient and task-relevant manner [51].

It has recently been suggested that cross-frequency coupling between the alpha (8–12 Hz) and gamma bands could implement a mechanism of pulsed inhibition by which salient stimuli are given priority over less salient ones [52]. This suggestion was inspired by evidence that the phase of alpha oscillations modulates both perception [53], [54] and gamma amplitude [18], [55]. Alpha activity likely serves to inhibit brain regions that are irrelevant to the behaviour at hand; e.g. to inhibit parts of visual cortex that correspond to non-attended parts of the visual field [56]. To still enable processing of important stimuli in those non-attended parts of the environment, by those inhibited brain regions, alpha activity creates a temporal code that serves to order the stimuli according to saliency. The actual processing itself is reflected in the gamma band, thus resulting in coupling between the phase of alpha and the amplitude of gamma [52].

While the present paper is mainly concerned with coupling between hippocampal theta and neocortical gamma oscillations, it seems equally applicable to such coupling within the neocortex between the alpha and gamma bands. The core findings of our model are upheld when we change the low-frequency signal from the theta to the alpha band, as shown in Figures S1 and S2. This is particularly interesting considering the crucial role we find for inhibitory cells in our model: the cross-frequency relation between alpha and gamma in humans is hypothesized to fulfill a temporal code in which local inhibition of brain regions is crucial. A mechanism of low-frequency modulation of gamma oscillations very similar to the one we explore here might thus elegantly explain the ordering and inhibition of stimuli according to saliency as well. Of course, further work is needed to determine to what extent this speculation holds.

In conclusion, our model demonstrates that the empirically observed coupling between the hippocampal theta and neocortical gamma rhythms crucially involves a neocortically local network of fast-spiking interneurons. Thus, our findings shed light on the mechanism behind large-scale cortical interactions responsible for an ‘oscillatory hierarchy’ of neuronal information processing [37], [50]. The crucial role of fast-spiking interneurons in cross-region phase/amplitude coupling is, we believe, an interesting and testable prediction of our model.

## Methods

All network simulations were conducted using version 7.1 of the NEURON simulation environment [34], released on January 15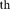, 2009. All data analyses were conducted using custom scripts, written either for NEURON 7.1, or for MATLAB (Mathworks Inc., Natick, MA , USA).

### Interneuron subnetwork

The interneuron subnetwork was modelled after some quite extensive previous neurophysiological and modelling work [20], [40], and consists of a virtual ring of 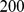 single-compartmental Hodgkin-Huxley-like model neurons [33]. We used this ring-like structure because we use a synaptic connection probability that is dependent on cell distance (described in more detail below): if we had just used an unfolded one-dimensional line, cells at the edges of the line would have fewer synaptic connections than cells near the middle of the line. Using a ring avoids this problem[20], [40].

Cells had a resting potential of 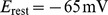 and a membrane surface area of 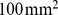. Leakage, Na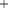, and K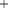 conductances were inserted into each neuron according to the model of Wang & Buzsáki ([25]; ‘ WB ’ conductances). These differ from standard Hodgkin-Huxley (‘ HH ’) conductances in two important respects (see appendix for detailed equations): first, the fast Na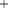 current activation variable 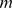 is substituted by its steady-state value 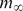; second, the remaining gating kinetics for the Na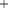 and K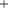 current are sped up by a factor 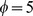. These specifics are computationally efficient, because of the 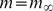 substitution, and, because of the adjusted time course of the currents, ensure that a firing pattern emerges that is characteristic of fast-spiking inhibitory interneurons [25].

Each interneuron receives incoming GABA -ergic synapses from a subset of its neighbours on the ring with a Gaussian probability dependent upon the distance between two cells, up to a maximum connection distance of 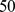 cells (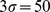). Synapses were either on or off; the Gaussian probability only governs whether or not there will be a synapse and is not used to determine synaptic strength.

Synaptic events were modelled by insertion of a conductance determined by a two-state kinetic scheme of the form:
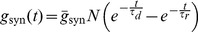
(1)with resulting current:
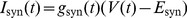
(2)where 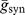 is the maximum synaptic conductance, 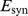 is the synapse reversal potential, and 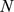 is a normalization factor:
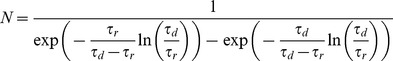
(3)that ensures that the peak conductance is given by the relevant parameter 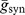.

The GABA -ergic synapses were governed by a rise time constant of 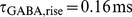 and a decay time constant of 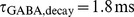. The unitary post-synaptic peak conductance was 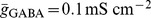. These values are consistent with empirical findings [57], [58]. The synaptic reversal potential was varied for the study of gamma rhythm generation, while it was set to 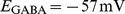 for all subsequent simulations, in accordance with gamma rhythm results and previous studies [28], [59].

In addition to GABA -receptors, AMPA - and NMDA -receptors were also present at the membrane of the fast-spiking cells, in order to model the connections between the two subnetworks and to model incoming spike trains as input. These were modelled by conductance insertion according to two separate bi-exponential functions of the type described by equation 1. Separate time constants were used to model the AMPA and NMDA currents: 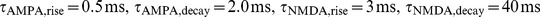 [60], [61]. The reversal potential for both receptor types was set to 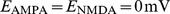. The unitary post-synaptic peak conductances for the glutamatergic synapses on the fast-spiking cells were given by 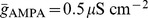 and 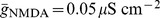. These conductance values were chosen to ensure that the average net current input to the fast-spiking cells, resulting from synaptic events, was comparable in amplitude to the direct current input used in the model of Vida and colleagues [28].

Apart from the above-mentioned chemical synapses, electrical synapses, or gap junctions, were included in the interneuron network. Gap junctions allow small quantities of ions to flow between two coupled cells; a given cell is thereby able to directly influence the membrane potential of another cell. Between each cell and its 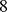 nearest neighbours, a gap junction was inserted with a probability of 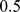, resulting in, on average, 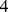 gap junctions per cell [28].

Gap junctions were modelled by a constant conductance insertion of 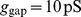 between two cells [62], [63]. The resulting current between two coupled cells 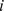 and 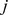 is given by
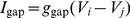
(4)


### Pyramidal cell subnetwork

The pyramidal cell subnetwork consists of a two-dimensional sheet of 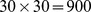 single-compartmental Hodgkin-Huxley model neurons (standard NEURON implementation) [33], [34]. This results in an anatomically realistic ratio of 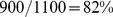 pyramidal cells versus 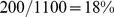 interneurons [13]. The resting potential of the pyramidal cells was equal to that of the interneurons, 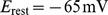, and standard Hodgkin-Huxley conductances were inserted to model cell membrane channels [33]. Each neuron received glutamatergic synaptic afferents from a subset of its neighbours according to a two-dimensional Gaussian probability, dependent on cell distance. Maximum connection distance was 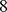 cells (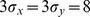), ensuring a realistic ratio of synaptic densities within the pyramidal cell subnetwork (see Table 1). The standard Euclidean measure was used to define the distance between cells:

**Table 1:** Model connectivity values.

	Model (%)	Anatomy (%)
FS 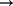 FS	4.24	1.34
P 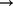 FS	4.47	4.97
P 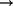 P	28.09	28.17
FS 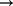 P	6.30	7.62
ext 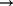 FS	7.09	6.66
ext 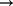 P	49.80	50.66

Shown is the number of synapses of different types, presented as ratios of the total number of synapses. Model values were either chosen to reflect connectivity known from anatomy (reported anatomical values are from [65]), or, in the case of the FS 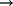 FS connectivity, based on previous modelling work [28].



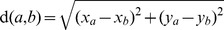
(5)As with the interneuron subnetwork, here it is also important to avoid edge effects on the number of synapses per cell. For this two-dimensional case, we created a torus out of our network by folding opposing edges onto each other:
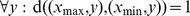


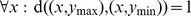



Incoming glutamatergic and GABA -ergic synaptic events were modelled by the same bi-exponential functions as described above for the fast-spiking cells (see equation 1), but with different peak conductances: for the pyramidal cells, 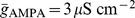, 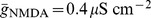, and 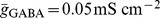. These values were chosen to obtain accurate cortical pyramidal cell firing characteristics for the model neurons; most importantly, they ensured an ongoing ‘background firing noise’ of about 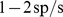 [13], [64].

### Subnetwork interconnections and network input

For the investigation of theta–gamma coupling, each pyramidal cell received GABA -ergic afferents from, on average, 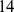 randomly chosen fast-spiking cells and each fast-spiking cell received glutamatergic afferents from, on average, 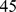 randomly chosen pyramidal cells. These values were chosen to obtain realistic ratios of synaptic densities between the two subnetworks (see Table 1). For the investigation of the influence of the gamma-synchronized interneuron subnetwork on the synchronization of the pyramidal cells, the average number of incoming P synapses per FS cell was kept constant at 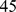, while the average number of incoming FS synapses per P cell was varied in the range between 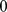 and 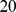.

For the investigation of gamma rhythm generation by the interneuron subnetwork, a direct current input was supplied to the fast-spiking cells. This allowed a straightforward comparison of our simulations with results found in the literature [28]. The amplitude of the current was different for different cells, and varied as a function of time. This resulted in a variable intrinsic firing frequency for each fast-spiking cell, enabling the study of the robustness of network synchronization as a function of different parameters.

Specifically, drive amplitude was determined by drawing a mean drive 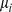 for each cell 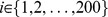 from a normal distribution with mean 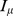 and then, for each time window of 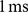, drawing a specific drive amplitude from a new normal distribution with mean 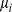:
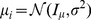
(6)

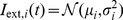
(7)


The spread of the distributions was defined in terms of coefficients of variation (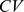), resulting in standard deviations 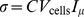 and 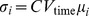. The variation over time was kept constant, 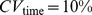, while both 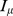 and 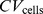 were varied to assess the network's robustness in generating a gamma rhythm.

To investigate (1) the influence of the gamma-synchronized interneuron subnetwork on the pyramidal cells and (2) the influence of the theta rhythm on the interconnected network of fast-spiking and pyramidal cells, direct current input was replaced by Poisson spike train input acting on glutamatergic synapses, to serve as a more realistic model of actual neuronal input.

Two pools of Poisson input spike trains were initialized, both consisting of 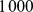 fibres. The fibres in the first pool had a constant average firing rate, 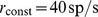, modelling cortical background noise. The firing patterns of these fibres were mutually uncorrelated. The fibres in the second pool varied their spiking rate according to a sinusoid, modelling the ascending fibres that carry the theta rhythm. The average spiking rate for the ‘variable-spiking’ fibres is given by:
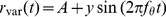
(8)with average spiking rate 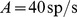, amplitude 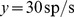, and frequency 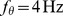. For each fibre in the two pools, the probability of a time window of 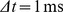 containing a single spike is given by 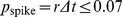.

Each pyramidal cell received incoming synapses from, on average, 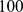 randomly selected fibres with a constant firing rate and 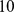 randomly selected variable-spiking fibres, while each fast-spiking cell received incoming synapses from, on average, 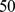 randomly selected fibres with a constant firing rate and 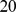 randomly selected variable-spiking fibres. The strong projection from hippocampal afferents to interneurons, relative to pyramidal cells, is in accordance with previous morphological and physiological findings ([31], [32]; see table 1).

All of the synaptic densities reported above (i.e., concerning synapses within one of the two subnetworks, synapses between the two subnetworks, and external afferent synapses) were either chosen to reflect anatomically known ratios of different synaptic types ([65]; see Table 1), or based on previous modelling work [28].

### Network analyses and simulation characteristics

#### Assessment of network synchrony

Two different measures were used to assess network synchrony. The first is the normalized averaged cross-correlation based ‘network coherence’ measure 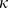 introduced by Wang & Buzsáki [25]. To determine this measure, two binary spike trains with bin size 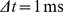 and resulting length 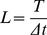 are given by 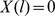 or 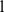, 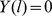 or 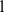, with 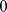 – no spikes and 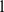 – a spike present in time bin with index 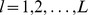. The pairwise coherence between two spike trains 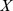 and 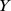 is given by:
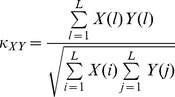
(9)


The network coherence measure 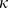 is then defined as the average pairwise coherence for all neuron pairs in the network. Wang & Buzsáki [25] define network coherence 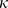 as a function of bin size and, indeed, this measure is quite strongly dependent upon bin size. Network synchrony is most accurately reflected by 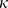, however, when bin size is small. As a statistic, therefore, we report this measure only for a bin size of 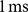, i.e., our 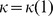. This is in accordance with previous work [40].

As a second measure of network synchrony, we introduce the average spike volley peak height. To automatically determine the occurrence of a volley peak, all of the network's spikes are first aggregated in 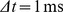 bins, such that the number of spikes in the interval 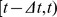 is given by 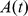 (time in ms). The occurrence of a peak is then defined as:
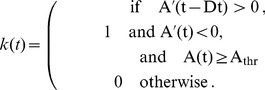
(10)where 
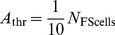
 for the fast-spiking cells and 
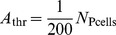
 for the pyramidal cells. The discrete derivative 
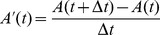
. The average spike volley peak height is given by:
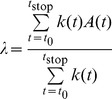
(11)with 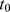 and 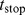 the begin and end times (in ms) of the simulation period to be analyzed, respectively.

#### Simulation timings

It is important to ensure that a truly (pseudo-)random firing pattern will emerge in the network, so that no initialization effects will influence the results. Therefore, at the beginning of a network simulation, at 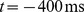, no synaptic connections are inserted, within or between the two subnetworks. At 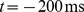, synapses and gap junctions are inserted, and all analyses of network firing characteristics are started at 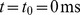, allowing the network to settle into its new, connected, state and preventing transient network properties due to synapse initialization to affect the results. All results reported in the present article were robust across simulations; i.e., the network always settled into the same state when initial conditions were identical. Noise due to the random number generator was of course different across simulations. For the study of gamma generation, simulations were stopped at 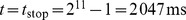; for the study of theta modulation of gamma oscillations, simulations were stopped at 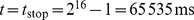.

#### Measure of electrical activity

The main phenomena in which gamma oscillations are usually said to occur are the local field potential (LFP) and electro-encephalogram (EEG). Both these measures of neural activity are thought to reflect the summed total electrical activity in the dendrites of pyramidal cells [see, e.g., 42] . In order to analyse not only the spike times, but also the continuous electrical activity of our network, we generated a simulated LFP by calculating the negative summed total pyramidal cell potential:
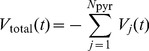
(12)and low-pass filtering this signal using an FFT -based finite impulse response filter with a 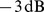 cutoff frequency of 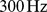. The LFP is taken as the negative, rather than the positive, summed total, because the LFP is measured *outside* the cells in electrophysiological work. The summed total pyramidal cell potential in our model corresponds to the potential *inside* the cells, so a sign change is needed for our simulated LFP to accurately correspond to its electrophysiological counterpart.

#### Assessment of theta modulation of spike times

To quantify the amount of theta modulaton of spike times in both the fast-spiking and pyramidal cell populations, we used two measures. To compute these, all of a subnetwork's spikes are first summed into 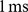 non-overlapping bins, such that the number of spikes in the interval 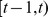 is given by 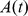 (time in ms). The resulting population activity measure is then aggregated over all theta periods:
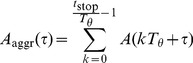
(13)with theta period 
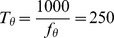
 and 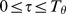.

The first measure used to quantify spike time theta modulation is the squared Pearson correlation coefficient 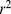, computed between this aggregate activity measure and the ‘raw’ theta signal given by:
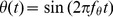
(14)


As a second measure of theta modulation of spike times, we introduce a simple *modulation score* 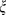. To compute this measure, the aggregate activity 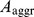 with bin size 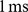 is re-binned into 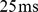 bins, resulting in an activity measure 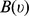 with 
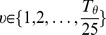
. This re-binning prevents any high-frequency (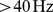) information from influencing the modulation score. The modulation score 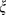 is then given by:
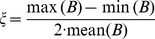
(15)and can be interpreted as the *relative amplitude* of any theta oscillation, if such an oscillation is reflected in the spiking activity of the network. The two subpopulations of cells, the FS and P cells, differ strongly in their average firing rate (as do pyramidal and fast-spiking cell populations in the real brain). Therefore, the absolute amplitude of theta-modulated spiking activity cannot be used to compare the two subpopulations. We thus use this relative amplitude measure 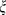, rather than an absolute amplitude, to compare the variations in activity in the two subpopulations of cells.

Note that, because of the period-wise aggregation described by equation 13, the time values 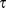 and 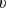 mentioned above correspond to certain phases 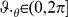 in the theta cycle:
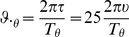
(16)


This relationship will be used in plotting spike counts versus theta phase.

#### Measure of cross-frequency phase/amplitude coupling

To quantify cross-frequency phase/amplitude coupling, we used a measure very similar to that employed by Canolty et al. [17].

First, a gamma-only signal is derived from the LFP by band-pass filtering the latter signal between 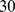 and 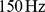. We denote this gamma-only signal by 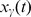. We obtain the analytic representation 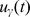 of 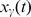 using the Hilbert transform:
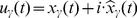
(17)

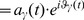
(18)where 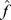 denotes the Hilbert transform of a function 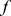, 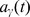 is the analytic amplitude time series, and 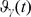 is the analytic phase time series. We also obtain the analytic representation 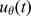 of the ‘raw’ theta signal (equation 14) used in determining the spike rates of the variable-rate input fibres:
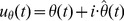
(19)

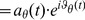
(20)


These two measures are combined into a single, complex-valued signal 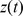:
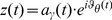
(21)


Theta phase is distributed uniformly, since the theta rhythm is generated according to a simple sine function. Therefore, if gamma amplitude and theta phase are independent, the distribution of 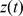 values should be approximately radially symmetric relative to the origin in the complex plane. If, however, the two are not independent, clustering of 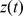 values in the complex plane is expected to occur. We use the distribution of 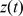 in the complex plane as a measure of theta phase/gamma amplitude coupling (see Figure 7).

Finally, note that our measure differs in an important respect from that used by Canolty et al. [17]. Canolty et al. 's measure was computed on two signals that were obtained by band-pass filtering the same signal using two different frequency bands. Our analysis uses a (gamma) band-pass filtered signal for the amplitude time series, but uses a different signal for the phase time series, namely the raw theta signal that governs the variable-rate input spike trains. This is simply an adaptation of Canolty et al. 's measure to a set of simulated data; the interpretation of the measure does not change.

## Supporting Information

Appendix S1 (PDF)

Figure S1 **Distributions of theta phase/gamma amplitude values for different frequencies of the theta signal provided as input to the model network.**
(EPS)

Figure S2 **Norm of the mean of the composite phase/amplitude distributions, as a function of theta frequency.**
(EPS)

Figure S3 **Norm of the mean of the composite phase/amplitude distributions, as a function of theta input amplitude, when only the pyramidal cells receive theta-modulated input.** The dotted line represents the value of this metric for the physiologically constrained parameters used in the original simulations.
(EPS)

Figure S4 **Correlations between the sinusoidal theta input signal, and the amplitude envelope of the LFP gamma oscillations, as a function of GABA-ergic synaptic reversal potential.** Since we know that GABA reversal potential greatly influences gamma synchrony in the interneurons, this parameter is here used as a proxy for manipulating gamma synchrony.
(EPS)

Figure S5 **Distributions of composite phase/amplitude values when using total postsynaptic current (right) versus membrane potential (left) as simulated LFP signal.**
(EPS)
