# Supplementary material for: Hippocampal Theta Modulation of Neocortical Spike Times and Gamma Rhythm: A Biophysical Model Study
Source: PLoS One. 2012 Oct 2;7(10):e45688. doi: 10.1371/journal.pone.0045688 (PMC3462809; doi:10.1371/journal.pone.0045688)
Supplement: Appendix S1 — (PDF) [file pone.0045688.s001.pdf]

Appendix: Detailed formulas

Fast-spiking cell membrane dynamics

The dynamics of the fast-spiking inhibitory interneurons were modelled according to previous work by Wang & Buzsáki [1]. The membrane potential for each fast-spiking cell is governed by the equation:

$$C_m \frac{dV}{dt} = -I_{\mathrm{Na}} - I_{\mathrm{K}} - I_{\mathrm{L}} - \sum I_{\mathrm{syn}} - \sum I_{\mathrm{gap}} + I_{\mathrm{ext}} \tag{1}$$

with membrane capacitance $C_m = 1\,\mu\mathrm{F\ cm^{-2}}$ and I_{syn}, I_{gap}, and I_{ext} as given by main text equations 2, 4 and 7, respectively. The leak, Na^+ and K^+ currents are given by:

$$I_{\mathrm{L}} = g_{\mathrm{L}}(V - E_{\mathrm{rest}}) \tag{2}$$

$$I_{\mathrm{Na}} = g_{\mathrm{Na}} m_\infty^3 h (V - E_{\mathrm{Na}}) \tag{3}$$

$$I_{\mathrm{K}} = g_{\mathrm{K}} n^4 (V - E_{\mathrm{K}}) \tag{4}$$

with conductances $g_{\mathrm{L}} = 0.1\,\mathrm{mS\ cm^{-2}}$, $g_{\mathrm{Na}} = 35\,\mathrm{mS\ cm^{-2}}$, and $g_{\mathrm{K}} = 9\,\mathrm{mS\ cm^{-2}}$ and reversal potentials $E_{\mathrm{Na}} = 55\,\mathrm{mV}$ and $E_{\mathrm{K}} = -90\,\mathrm{mV}$. The gating variables m_∞, h, and n are governed by:

$$m_\infty = \frac{\alpha_m}{\alpha_m + \beta_m} \tag{5}$$

$$\frac{dh}{dt} = \phi(\alpha_h(1-h) - \beta_h) \tag{6}$$

$$\frac{dn}{dt} = \phi(\alpha_n(1-n) - \beta_n) \tag{7}$$

with $\phi = 5$ and the following α and β functions:

$$\alpha_m(V) = \frac{-0.1(V+35)}{\exp(-0.1(V+35))-1} \tag{8}$$

$$\beta_m(V) = 4\exp(-(V+60)/18) \tag{9}$$

$$\alpha_h(V) = 0.07\exp\left(-\frac{V+58}{20}\right) \tag{10}$$

$$\beta_h(V) = \frac{1}{\exp(-0.1(V+28))+1} \tag{11}$$

$$\alpha_n(V) = \frac{-0.01(V+34)}{\exp(-0.1(V+34))-1} \tag{12}$$

$$\beta_n(V) = 0.125\exp\left(-\frac{V+44}{80}\right) \tag{13}$$

References

1. Wang XJ, Buzsáki G (1996) Gamma oscillation by synaptic inhibition in a hippocampal interneuronal network model. Journal of Neuroscience 16: 6402–6413.
